# Discovery of novel genetic syndromes in Latin America: Opportunities and challenges

**DOI:** 10.1590/1678-4685-GMB-2023-0318

**Published:** 2024-03-11

**Authors:** Víctor Faundes, Gabriela M. Repetto, Leonardo E. Valdivia

**Affiliations:** 1 Universidad de Chile, Instituto de Nutrición y Tecnología de los Alimentos, Laboratorio de Genética y Enfermedades Metabólicas, Santiago, Chile.; 2 Universidad del Desarrollo, Facultad de Medicina, Instituto de Ciencias e Innovación en Medicina, Centro de Genética y Genómica, Programa de Enfermedades Raras, Santiago, Chile.; 3 Universidad Mayor, Facultad de Ciencias, Centro de Biología Integrativa, Santiago, Chile.; 4 Universidad Mayor, Facultad de Ciencias, Escuela de Biotecnología, Santiago, Chile.

**Keywords:** Bioinformatics, gene discovery, genomics, model organism

## Abstract

Latin America (LatAm) has a rich and historically significant role in delineating both novel and well-documented genetic disorders. However, the ongoing advancements in the field of human genetics pose challenges to the relatively slow adaption of LatAm in the field. Here, we describe past and present contributions of LatAm to the discovery of novel genetic disorders, often referred as novel gene-disease associations (NGDA). We also describe the current methodologies for discovery of NGDA, taking into account the latest developments in genomics. We provide an overview of opportunities and challenges for NGDA research in LatAm considering the steps currently performed to identify and validate such associations. Given the multiple and diverse needs of populations and countries in LatAm, it is imperative to foster collaborations amongst patients, indigenous people, clinicians and scientists. Such collaborative effort is essential for sustaining and enhancing the LatAm´s contributions to the field of NGDA.

## Introduction

### Brief history of gene-disease association discovery

Several monogenic disorders (genetic syndromes hereafter) have been described throughout human history, even before the birth of genetics as a discipline. Achondroplasia (Kozma, 2006), Marfan syndrome (Schoenfeld, 1978) or haemophilia A (McKusick, 1965) are examples of inherited disorders that were first described several centuries ago, but only during the 20^th^ century a genetic cause was identified due to both scientific and technological advances and to the access to vast amounts of genotype and phenotype data. To date, there are almost 5000 protein-coding genes whose allelic variation causes Mendelian disorders in Online Mendelian Inheritance in Man (OMIM) (Amberger et al., 2019; Antonarakis, 2021).

The elucidation of exact mechanisms on how DNA sequence variants influence genetic disorders is intrinsically linked to technological developments, resource availability, and population studies. From the chain-termination method of DNA sequencing invented by Frederick Sanger in 1977 to current long-reads or single-cell sequencing technologies, the field of medical genetics has witnessed an explosion in the identification of novel disorders (Claussnitzer et al., 2020). This technological development also contributed to a large amount of novel questions and uncertainties, such as the thousands of variants of uncertain significance [VUS] that a single whole-genome sequencing test can unveil (Rehm et al., 2023). These uncertainties are being solved currently thanks to the parallel development of publicly available resources such as ExAC (Lek et al., 2016) and subsequently gnomAD (Karczewski et al., 2020), or ClinVar (Landrum et al., 2016). The advent of comprehensive population studies that apply cutting-edge sequencing technologies coupled to deep and systematic phenotyping such as the UK BioBank Project (Sudlow et al., 2015), also contributes to this virtuous circle of technologies, resources and population analyses that fuel the understanding of medical genetics. Moreover, the use of model organisms to assay pathogenicity of newly found variants leads to the exploration of underlying mechanisms of pathophysiology. This has also catalysed the discovery of novel drug targets to tackle both rare and common inherited disorders (Claussnitzer *et al.*, 2020; Baldridge et al., 2021). 

The field of genetics is in constant evolution. Remarkably, LatAm has a unique genetic heritage with admixture from African, European, and Native American ancestral populations, providing a valuable resource to gauge how local genetic variants modulate phenotypic diversity in health and disease. Therefore, it is important to review the contribution that scientists in LatAm have made to delineating genetic syndromes in the past, as well as the current efforts and the prospects that LatAm can offer to the rest of the world in this field.

### Past and present contributions of Latin America to delineation of genetic syndromes

**Figure 1 -:**
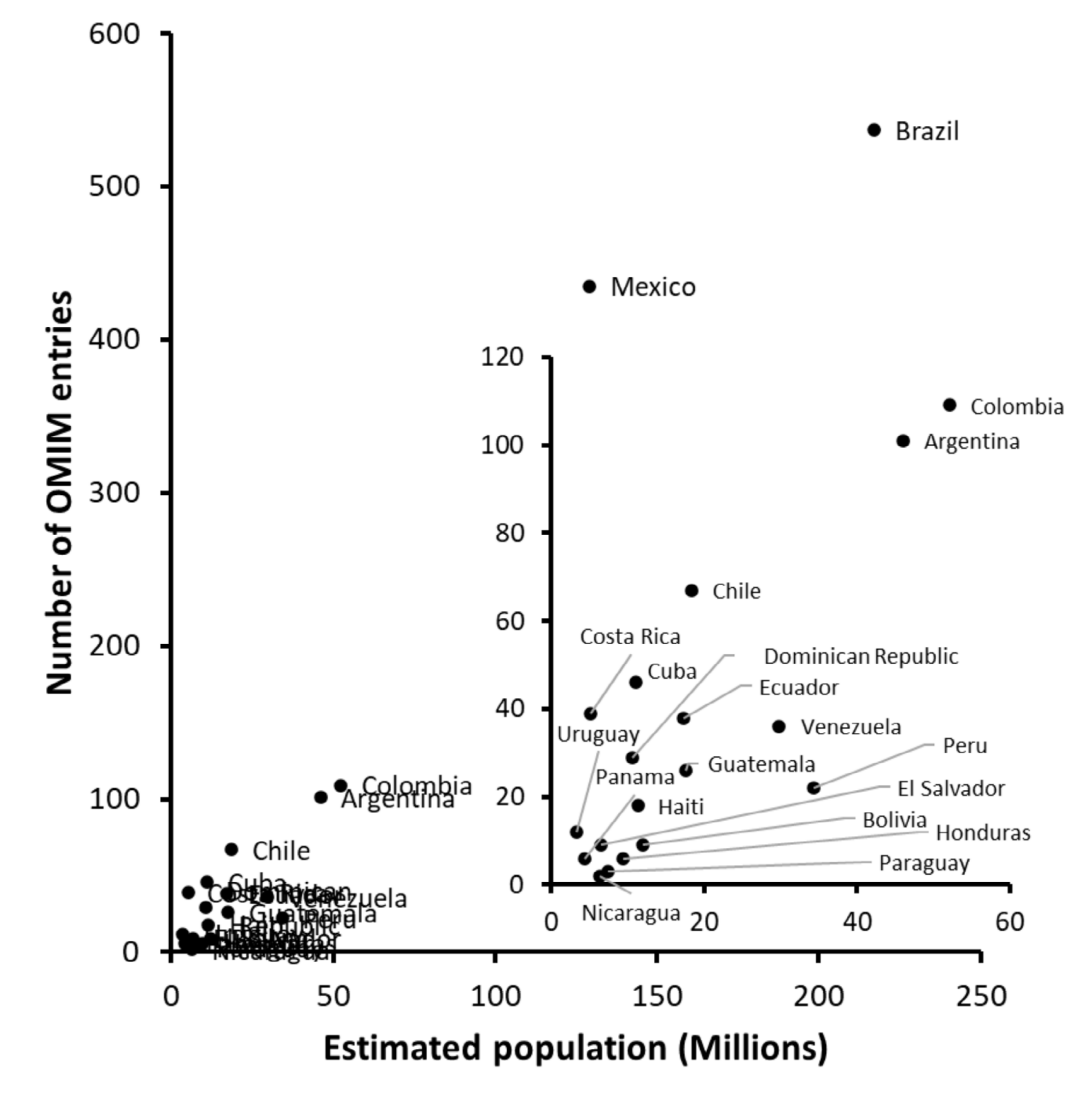
Correlation between estimated population of Latin American countries and their mentions in OMIM entries. The population estimations were obtained from wikipedia.org, which in turn obtained those numbers from international or national official organisations, whereas the OMIM entries were searched using the terms “country name” OR “its demonym” (e.g. Argentina OR Argentinian). The results were retrieved from both sources on 9th June 2023.

Medical genetics in LatAm has evolved over time (Penchaszadeh, 2004). Despite some known challenges such as the underrepresentation of LatAm people in genomic studies, local scientists have characterised new or known syndromes with confirmed or suspected Mendelian inheritance (Figure 1). There is a correlation between the current estimated population size of every country with the number of entries in the Online Mendelian Inheritance in Man Database (OMIM) that mentions a given country or its demonym (*s*=0.738; p=2.026×10^-4^). As expected, Brazil has contributed the most, whereas Nicaragua the least to those entries, in proportion to their populations (Figure 1). 

LatAm has contributed with world-renowned medical geneticists throughout time. These include the Brazilians Antonio Richieri-Costa and Maria Leine Guion-Almeida, who described new syndromes and delineated many others (Richieri-Costa and Guion-Almeida, 1992; Guion-Almeida *et al.*, 2006; Gil‐da‐Silva‐Lopes, 2019); the Argentinian Eduardo E. Castilla, who started the Latin American Collaborative Study of Congenital Malformations (ECLAMC), a pioneering, observational, prospective study on congenital malformations across the continent (Cavalcanti, 2018); the Mexican José María Cantú, who delineated several syndromes associated with camptodactyly and the syndrome with his name (Penchaszadeh et al., 2014); and Luis Morquio, who delineated Mucopolysaccharidosis Type IVa for first time almost a hundred years ago (Baujat and Valayannopoulos, 2014). More recently, the contribution of thousands of Venezuelans allowed mapping and subsequent discovery of the *HTT* gene, responsible of Huntington’s disease, where the contribution of Venezuelan geneticist Ernesto Bonilla was crucial (Gusella et al., 1983; The Huntington’s Disease Collaborative Research Group, 1993). These works were also relevant as they opened the research field of novel gene-disease associations (Table 1).

**Table 1 -:** Past and current contributions of Latin American geneticists to the discovery of novel genetic syndromes.

Syndrome (OMIM #)	Geneticist (Country of Origin)	Year of Publication	Studied Population	References
Mucopolysaccharidosis Type IVa (253000)	Luis Morquio (Uruguay)	1929	Uruguayan	(Baujat and Valayannopoulos, 2014)
Cantu syndrome (239850)	José María Cantú (Mexico)	1982	Mexican	(Cantu et al., 1982)
Huntington’s disease (143100)	Ernesto Bonilla (Venezuela)	1983 and 1993	Venezuelan	(Gusella et al., 1983; The Huntington’s Disease Collaborative Research Group, 1993)
Richieri-Costa/Guion-Almeida syndrome (268850)	Antonio Richieri-Costa and María Leine Guión-Almeida (Brazil)	1992	Brazilian	(Richieri-Costa and Guion-Almeida, 1992)
Steel syndrome (615155)	Claudia Gonzaga-Jauregui (Mexico)	2015	Puerto Rican	(Gonzaga-Jauregui et al., 2015)
Faundes-Banka syndrome (619376)	Víctor Faundes (Chile)	2021	Multi-ancestry	(Faundes et al., 2021)
Radiohumeral fusions with other skeletal and craniofacial anomalies (614416)	Denise P. Cavalcanti (Brazil)	2023	Multi-ancestry	(Silveira et al., 2023)

The comprehensive clinical depiction done by these and many other clinical geneticists, albeit always necessary, has moved in a reverse way in the current genomics era (“reverse phenotyping”, see below). Despite some difficulties, novel syndromes are being discovered from exome or genome analyses in LatAm individuals, with significant contributions from local scientists and clinicians (Vishnopolska et al., 2018; Coelho et al., 2022). Indeed, the discovery of the faulty gene in Steel syndrome (Gonzaga-Jauregui et al., 2015) or the better delineation of a poorly understood skeletal dysplasia (Silveira et al., 2023) are good examples of the potential that genomic research lead by LatAm scientists and considering LatAm participants can have (Table 1).

At the population level, big efforts are underway in several LatAm countries to describe genomic variants across thousands of individuals. By deploying genome-wide sequencing applications (Borda et al., 2023; Ziyatdinov et al., 2023), it is now possible to characterize the genetic architecture of the highly admixed LatAm population. This is one of several critical steps to continue unveiling novel genetic syndromes with global impact in the 21^st^ century.

## How are novel gene-disease associations discovered nowadays?

### An overall workflow

Historically, genetic syndromes have been “discovered” by identifying a set of individuals who share clinical features that did not match to one of the known contemporaneous syndromes, and had evidence to suspect a Mendelian inheritance pattern. Subsequent genetic analyses in those individuals led to the identification of variants in a specific gene-disease associations (GDA), which were statistically and/or functionally tested to demonstrate their definite causality. This classical workflow has been named as “forward phenotyping” (Swietlik et al., 2020), similar to “forward genetics” approaches used by animal model geneticists, who screened for phenotypes in mutagenized animal stocks (Irion and Nüsslein-Volhard, 2022). Albeit successful in identifying many GDA, its rate of discoveries was somewhat slow (Bamshad et al., 2019; Claussnitzer et al., 2020). 

**Figure 2 -:**
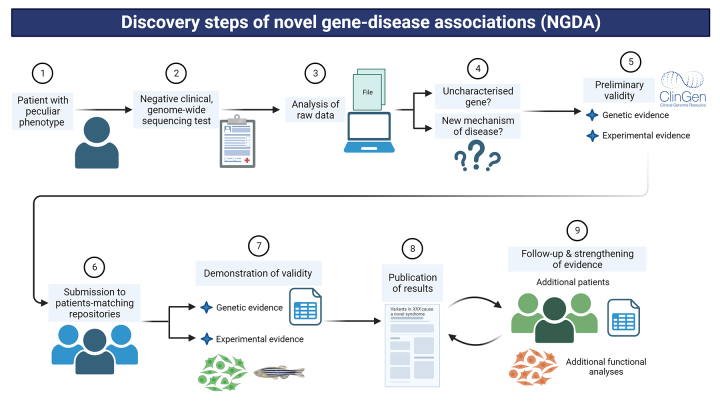
Steps currently performed for the discovery of novel gene-disease associations. The workflow of discovery of novel genetic syndromes is summarised in 9 basic and critical steps, from the evaluation of an index patient to the confirmation of the novel condition over time, considering how this type of research is performed nowadays with current techniques. 1) Clinical evaluation and requisition of genomic test, 2) return of negative results in already-associated genes, 3) analysis of raw data focused on unreported genes/variants, 4) identification of candidate variant(s)/gene according to metrics, 5) checking of previous findings using ClinGen criteria, 6) submission of clinical and genetic data to repositories, 7) comparison of clinical data amongst patients and development of functional studies, 8) publication of NGDA, and 9) expansion of evidence with further cohorts and functional data. Created with BioRender.com.

The development of genomic techniques, which allow comprehensive studies of all genes or even the whole genome, has allowed to search for a faulty genotype in patients with an unclear phenotype in a hypothesis-free manner, i.e., not led by the suspicion of a specific genotype (Gonzaga-Jauregui and Zepeda Mendoza, 2021). This configuration starts with the identification of a specific, suspected genotype in one affected individual by an uncharacterized syndrome, and the subsequent overlapping of clinical findings with other individuals with a similar genotype then can confirm a novel GDA (NGDA). This process has been named “reverse phenotyping”, and is one of the critical steps for the discovery of novel genetics syndromes nowadays (Figure 2) (Bamshad et al., 2019; Claussnitzer et al., 2020; Swietlik et al., 2020; Wilczewski et al., 2023).

Currently, the process of discovery of NGDA is as follows (Figure 2). A patient with congenital anomalies and/or neurodevelopmental disorders is evaluated by a medical geneticist (Step 1), who may have or not a specific suspicion. Then, a genome-wide sequencing approach such as a gene panel, exome or genome sequencing is requested to confirm a diagnostic hypothesis or search for an unsuspected genetic aetiology. The clinical report may not reveal any relevant variant in known, associated genes so far, or may reveal a VUS whose mechanism of disease, if any, is not completely understood (e.g. a protein-truncating variant is detected in a haploinsufficiency-intolerant gene, but which is currently associated with a disorder caused by gain-of-function or dominant negative variants in it) (Step 2). This should lead to the review of raw data if clinical scientists are trained to do so (Step 3). Depending on the approach, two basic questions must be raised after the new filtering process of raw data (Step 4): is there a relevant variant in an as-yet non-associated gene? Is the variant located in a known gene but it suggests a novel mechanism of disease? If the answer is yes for one of these questions, then it is possible that the patient has a novel genetic syndrome. This hypothesis must be preliminary tested using the Clinical Genome (ClinGen) Resource framework criteria that was built for this purpose (see below) (Step 5). 

Following the above mentioned workflow, it is not necessary to initially perform experimental analyses but rather to comprehensively review the data available about the gene and the variant(s) following the ClinGen criteria. Then, it is necessary to try to solve the following questions: is the gene tolerant or intolerant to some type of variant according to published metrics (e.g. gnomAD’s pLI or Z scores (Karczewski et al., 2020), GeVIR (Abramovs et al., 2020) or CCR (Havrilla et al., 2019) constraints, etc)? Is(are) the variant(s) seen in control databases (e.g. gnomAD (Karczewski *et al.*, 2020), 1000 Genomes Project (Genomes Project *et al.*, 2015), etc.)? Do(es) the variant(s) match(es) the expected inheritance and/or segregation? Are there cases with similar genetic and clinical findings published in the literature or patient databases (e.g. DECIPHER (Foreman et al., 2022), ClinVar (Landrum et al., 2016), etc.)? Is (are) the variant(s) located in a critical region/domain of the protein according to UniProt (UniProt, 2023) or may it lead to nonsense-mediated decay? Are gene expression, function and interactions concordant with those clinical findings according to current knowledge (e.g that deposited in UniProt, GTEx Project (GTEx Consortium, 2015), STRING (Szklarczyk et al., 2015), etc.)? Finally, is there evidence in model organisms about the consequences of gene dysfunction that are comparable to some of the clinical features found in affected patients, that can be found in databases such as ZFIN (Howe et al., 2013) or IMPC (Koscielny et al., 2014)? 

If the compiled evidence strongly suggests that the detected genotype in that patient is not random but possibly causal, then it is worth to submit the case’s phenotype and his/her variant(s) to patient-matching repositories (Step 6, see below) to connect with the global community and perform reverse phenotyping with clinically similar individuals. If reverse phenotyping shows some overlapping amongst the patients, then the formal process of demonstrating causality starts, considering the criteria that ClinGen evaluates at the moment of curating NGDA in order to plan next research phases (Step 7, see below). This step relies on several critical assumptions: equitable collaboration that requires data sharing, sufficient funding, laboratory resources, timely ethical review, amongst others, and may be also one of the longest ones and it may take several years to reach confident results. After consistent results are obtained, they should be published in both clinical repositories of variants (e.g. ClinVar) and high-impact journals (Step 8) in order to share the discoveries and help other clinicians and laboratories to understand similar findings in their patients. This subsequent collaboration is crucial to strengthen this NGDA, and to unveil further clinical and pathophysiological consequences of the novel genetic syndrome in humans (Step 9), which may lead to propose a modifying treatment and better follow-up, for example.

### The Clinical Genome (ClinGen) Resource framework

The state-of-the-art in DNA sequencing, bioinformatics, and data sharing have led to the discovery of a growing number of genetic variants that cause human diseases. Hundreds of novel congenital conditions have been discovered, and those discoveries have improved our understanding of the relevance in human development and physiology of hundreds of genes when affected by germline variants (Bamshad et al., 2019). Albeit many genes have been found causal of monogenic disorders when mutated, the quality and quantity of the evidence that support such causalities are very heterogeneous. Thus, the first attempt to standardise the causality of certain phenotype by variants in novel genes emerged in 2014, although it did not organise the evidence in levels to guide the process (MacArthur et al., 2014).

In 2017, ClinGen created and published a semiquantitative framework that allows to classify gene-disease associations by both the quantity and quality of the evidence that supports or refutes such causality. This framework is intended for known or novel genes that cause monogenic disorders when altered, following autosomal dominant, autosomal-recessive, or X-linked inheritance, but not for polygenic or multifactorial diseases (Strande et al., 2017). These guidelines have helped clinicians in the process of interpreting genetic test results for clinical care, and genetic laboratories to develop or interpret gene panels and genome-wide sequencing tests (Strande *et al.*, 2017; Thaxton et al., 2022). This framework has been adopted internationally and currently several groups of scientists work on curating these findings following its rules and posting the list of genes, their causalities over human phenotypes, and the level of evidence that support or refute that causality (DiStefano et al., 2022). Albeit the scientific team involved in the discovery of a particular new genetic syndrome do not do the curation *per se*, it is relevant it follows the criteria suggested by ClinGen at the moment of performing the research in order to get strong evidence of causality.

According to the ClinGen 2017 guidelines, there are two types of evidence to support that a gene causes a monogenic disorder when altered in the germline, i.e., constitutionally: genetic and experimental, which can add up to 18 points in total score. The genetic evidence can provide up to 12 points and is organised in two subtypes depending on the source of data: compilation of cases with genetic and phenotypic similarities, or case-control studies with statistical significance. The experimental evidence can add up to 6 points and is organised in three subtypes depending on the function of the gene (including expression and interactions), how the detected variants alter its cellular function(s), and if the phenotype can be replicated in a model organisms such as mouse, zebrafish, or other when the variants are engineered in the corresponding ortholog gene (Strande et al., 2017). Based on the sum of points obtained by the scientific curator team, gene-disease causalities may be classified as limited (1-6 points), moderate (7-11 points), strong (12-18 points), or definitive (12-18 points and extra patients with similar genetic and clinical findings are reported over time). Although it is not required both types of evidence to meet a minimum range of points to propose a NGDA, actually both are essential to demonstrate causality when curated. Indeed, >150 genes have been disputed or even refuted to cause their corresponding disease when mutated, which means that many patients were told to have a specific genetic disease that is not supported scientifically due to contradictory data (Strande *et al.*, 2017; DiStefano et al., 2022). 

### Patient-matching repositories

Patient-matching repositories are primarily scientific, online resources that allow connections amongst patients, their families, clinicians and researchers from around the world who share an interest in the same gene, because those patients have one or two variants of interest in that posted gene and some manifestations. To date, there are several of these repositories, albeit one of the most famous and used one is GeneMatcher (Sobreira et al., 2015), which is part of the Matchmaker Exchange Project (Philippakis et al., 2015), an initiative that combines 8 of these platforms. These repositories have eased and accelerated the discovery of novel syndromes caused by genetic changes in as-yet-unreported genes, allowing to diminish the diagnostic odyssey and democratising access to scientific research without requirements of large cohorts, which is crucial for rare disorders. (Boycott et al., 2022; Hamosh et al., 2022). This means that a clinical geneticist or researcher does not need to see all the patients that are being recruited, as that part is done by other clinicians, and the data is shared and stored electronically, facilitating reverse phenotyping. This way of collaborative phenotyping has not only accelerated the process of discovery of novel genetic syndromes, but it has also helped to expand the phenotypic spectra of both known and unknown genetic syndromes. It also has eased the study of *ex-vivo* functional mechanisms of disease, i.e. to build experimental evidence from patients’ samples with common genetic findings (Wilczewski et al., 2023). This type of experimental data is also considered by ClinGen as evidence to confirm or refute a NGDA, and it is at the same time a good way to accelerate the discovery of disease-modifying drugs. 

### The use of model organisms

In many cases that undergo clinical or research next-generation sequencing, the variant(s) in the responsible gene can be identified, but the mechanism of disease cannot be easily deducted. This diagnostic challenge (that can be critical for novel genetic syndromes) may be solved using model organisms: they can also help to discover novel gene-disease correlations, inform pathogenicity of DNA variants, and reveal underlying pathophysiological mechanisms with the potential of suggesting new therapies (Baldridge et al., 2021). The Model Organism Screening Center (MOSC) of the Undiagnosed Disease Network (UDN) is an example of a unique initiative that utilizes human genomic data to drive functional studies in model organisms seeking to aid in genetic diagnosis. The MOSC provides a framework for collaboration of basic scientists and clinicians to drive effective diagnoses. Customized experimental plans take into account a patient presentation, genes and variants, and appropriateness of a model organism analysis (worm, fly, or zebrafish). Remarkably, the MOSC has value beyond individual patients because both the new disease gene discoveries and phenotypic expansions accelerate diagnoses of additional patients who may have the same genetic condition, thereby reducing costs for many families (Baldridge et al., 2021).

## Opportunities and challenges for novel gene-disease association discovery in Latin America

### Opportunities

**Figure 3 -:**
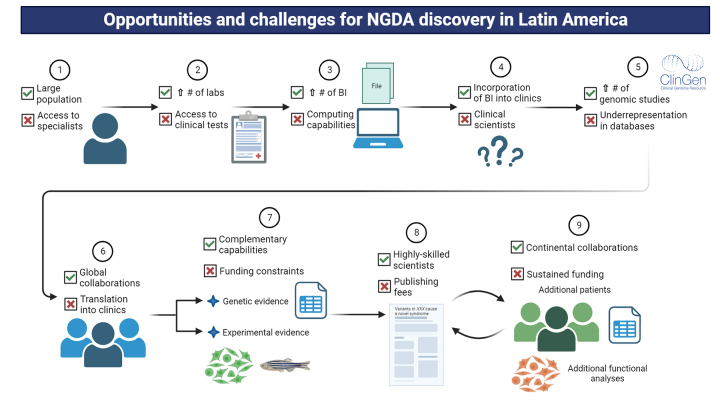
Opportunities and challenges that LatAm has to contribute to the discovery of NGDA. The opportunities (denoted with a green tick) and challenges (denoted with a red cross) of the continent are summarised in the same steps described for the discovery of NGDA workflow. Abbreviations and symbols: BI=Bioinformatics/Bioinformatician, #=Number, ↑=Increase. Created with BioRender.com.

LatAm has many comparative advantages and peculiarities that make it a potential game-changer in the field of medical genetics and genetic syndromes discovery, continuing the path that geneticists started decades ago and several are following currently using cutting-edge techniques, which are depicted in Figure 3., The continent has opportunities and strengths in every of those NGDA discovery steps.

The estimated current population in LatAm is about 650 million people (Step 1, Figure 3). The population is highly heterogeneous considering multiple ancestries and migration processes the number of distinct Amerindian groups, and the endogamy that some populations have experienced due to geographical isolation (Homburger et al., 2015; Bronberg et al., 2016; Norris et al., 2018). Thus, there may be “natural human knockouts”, i.e. people with bi-allelic, loss-of-function variants in as-yet unknown genes, which may have peculiar physiological effects and eventually lead to rare autosomal recessive disorders (Alkuraya, 2015; Saleheen et al., 2017). Also, this large population increases the likelihood that individuals have *de novo*, heterozygous variants leading to dominant disorders not yet described (Kaplanis et al., 2020). Therefore, it is plausible that this understudied population may have novel genetic syndromes, with potential global impact, that are awaiting to be discovered.

There is a growing number of clinical laboratories applying massive parallel sequencing (Step 2, Figure 3), either with their own sequencers or outsourcing the wet process to third parties (Torres et al., 2017; Alvarez-Gomez et al., 2021). Albeit it is difficult to estimate an exact number of laboratories, it is plausible to think that the current number of projects and organisations involved in studying human genomics translate the country’s sequencing capabilities, which in turn may have a some correlation with each country’s population (Brazil has 9 of those projects and organisations, Mexico 8, Colombia 8, Argentina 7, Chile 7, Costa Rica 8) (see “Internet Resources” section, “World of Genomics” for further details). This allows that many more individuals can be diagnosed with a genetic disorder or be subject to further analyses for discovery of NGDA. Linked to these capabilities, several LatAm universities have raised strong research groups in bioinformatics and the number of articles published in the area is growing (De Las Rivas et al., 2019). Also, some LatAm universities are partnering with the European Bioinformatics Institute to grow both the capabilities, resources and training in bioinformatics through the CABANA Project (Stroe, 2022) (Step 3). LatAm hospitals and healthcare centres are including electronic health records and data science as part of their healthcare policies (Step 4) (Tejerina et al., 2020; Rosa and Frutos, 2022), which eases genomic analyses. Although it is not clear if bioinformaticians are being recognised as healthcare workers at LatAm hospitals, the clinical implementation of genomic programmes at these places suggest that their work is being recognised (Torres *et al.*, 2017; Alvarez-Gomez *et al.*, 2021), and therefore, it highlights their relevance for the healthcare system. Thus and considering all aforementioned steps, the likelihood of unmasking the causal genotype in an affected LatAm person is increasing.

In addition to the increasing availability of clinical laboratories performing genomic tests, there is a growing number of research projects that involve the study of hundreds to thousands of patients with rare diseases, which are identifying NGDA in LatAm population. The Brazilian Rare Genomes Project (Coelho et al., 2022), “Decoding Complex Inherited Phenotypes in Rare Diseases” Project in Chile (Poli et al., 2024) or the Mexican Network of Rare Diseases (https://enfermedadesraras.liigh.unam.mx/) are some examples that can contribute to discovery of novel genetic syndromes (Step 5, Figure 3). These projects are led by a new generation of reputed geneticists with a broad expertise in the field of NGDA and who collaborate with international consortiums and organisations (e.g. Global Genomic Medicine Consortium [G2MC], International Rare Diseases Research Consortium [IRDiRC]) (Step 6). This promotes a culture sharing and global discussion of cases with novel findings, and reduces the testing gap in LatAm individuals. The results of these projects and collaborations can be relevant to the design of healthcare policies in genomic medicine (or for rare disorders).

The discovery of NGDA requires research laboratories and trained scientists in LatAm to generate the experimental evidence needed to support discoveries. Local laboratories have the capabilities for developing classical techniques such as Western blot, RT-qPCR, microscopy analyses, the use of model organisms, amongst many others. For more modern, complex techniques, LatAm scientists are increasingly being funded for the acquisition of tools that allow massive and complex analyses such as confocal microscopes, single-cell sequencing devices, mass spectrometers and many others (Step 7, Figure 3) (Arnold, 2023). Of note, the applications for equipment funding require that applicants are highly-skilled, able to handle them, and ideally have previous experience on manipulating those devices. Therefore, LatAm has the required scientists to perform both classic and cutting-edge experiment to get the necessary experimental evidence, and to publish it in reputed journals (Step 8) (Arnold, 2023). Finally and recognising the similar challenges that LatAm countries face, several continental collaborations are emerging such as the Genetics of Latin American Diversity (GLAD) Project (Borda et al., 2023) or the COVID-19 Regional Genomic Surveillance Network (COVIGEN) (Leite et al., 2022), which are allowing to share and delineate genomic findings in our continent, inform causality or predisposition to diseases, and joining forces for a common purpose (Step 9).

### Challenges

LatAm has historically faced many challenges and threats that have precluded its scientific development at the same level and speed than high-income countries. Considering the steps of discovery of NGDA (Figure 3), this part of the continent should address the following challenges in order to exploit its potential and strengthen its role as a crucial place to deepen the knowledge on medical genetics.

The current access to the specialised care that is required for NGDA is limited in most countries of LatAm. Access to healthcare centres and specialists that allow the initial suspicion of a genetic syndrome is affected by both the absolute number of trained clinicians and the unequal distribution of them even within the same country (Step 1, Figure 3) (Giugliani et al., 2022). In line with the limited human resources, most individuals and families cannot afford the current price of genomic tests, and very few LatAm countries have considered the financial coverage of them as a universal policy (Step 2) (Encina et al., 2019; Alvarez-Gomez et al., 2021; Giugliani *et al.*, 2022). Also, it is still very expensive to implement a genomic laboratory in LatAm due to 1) high cost of reagents and machinery, 2) inexistence of reagents/machinery manufacturers in the continent and high delivery costs from the Global North, 3) high import taxes, 4) lack of technical support in the region, amongst others, which constitute major bottlenecks for the greater implementation of genomic sequencing in LatAm for both clinical and research purposes (Step 2). In addition to the sequencing capabilities, powerful data storage and informatics units are required for constant re-analyses of growing genomic data, which are lacking in current LatAm hospitals (Step 3) (Tejerina et al., 2020). Lastly, and as part of the limited resources of the LatAm healthcare systems, there are still very few clinicians that hold a PhD or equivalent degree and training to perform/supervise both the basic and clinical research that is required for NGDA (Coronel et al., 2011; Labbe Atenas et al., 2017; Ríos et al., 2019). Hospitals do not consider specific incentives for clinicians or healthcare workforce to perform clinical or translational research as part of their contracted duties (Step 4) (Ciocca and Delgado, 2017). Therefore, and considering current approaches, LatAm healthcare systems have several limitations to even suspect a novel genetic syndromes.

Some global problems, not only restricted to LatAm, may affect the rest of the continent with even a stronger impact. It is well known that many ancestries are underrepresented in genomic databases and studies. Considering the heterogeneity of the LatAm population, the continent is especially sensitive to this (De Ver Dye et al., 2021; Borda et al., 2023). The gap is critical when preliminary analyses of the ClinGen criteria are applied, especially when an inherited but undescribed variant is detected in a candidate gene in databases as it is not possible to discard or consider it with confidence for further analyses (Step 5, Figure 3). Also, although patient-matching repositories have demonstrated to be very useful for research purposes, their clinical utility has not been fully explored and how this may impact clinical management and surveillance (Boycott et al., 2022; Hamosh et al., 2022). For example, for known, already-associated genes, patients with clinically relevant variants (either pathogenic or VUS) are not usually submitted to those platforms. This information could guide the interpretation of the variant, lead to propose possible phenotype expansions, or help delineate other examinations the patient may require (Step 6). Thus, some global uncertainties are very relevant for NGDA in LatAm and require further efforts for data sharing along publication of local genetic features.

Finally, the lack of constant and substantial research funding is probably the most relevant challenge to experimentally support a NGDA (Step 7), openly publish the results (Step 8), and expand the knowledge of a genetic syndrome considering LatAm cohorts with further experimental analyses, especially with the use of model organisms (Step 9). This limitation is explained, in part, by several factors. First, the percentage of gross domestic product for research purposes in LatAm countries is low when compared to developed countries (Coronel et al., 2011; Ciocca and Delgado, 2017). Second, there are no specific grants for research in rare diseases and genetic syndromes (Encina et al., 2019; Wainstock and Katz, 2023). And third and because research on rare diseases faces many challenges such as sample size, unknown prevalence and natural history, etc. that are not understood/known by review panels, and therefore research projects in this field are not usually funded (Institute of Medicine, 2010). Consequently, most NGDA projects continue to be led by scientists from developed countries with LatAm scientists mainly contributing phenotypic data and samples from few patients for the clinical delineation of a novel genetic syndrome, in what can be considered “science-by-email or courier”. Finally, the lack of updated regulatory frameworks and their heterogeneity across the continent may delay and even stop the implementation and development of genomics in the region, not only for diagnostic purposes, but also for therapeutic ones in each of the aforementioned steps (Rozas et al., 2022).

## Discussion

LatAm is a region with an exceptional potential for NGDA, not only in terms of its highly heterogeneous, large and underexplored population, but also because of its growing genomic capabilities and highly trained researchers. However, it is necessary to accelerate the implementation of genomics into clinical practice to decrease the current challenges and gaps as recently recommended by the World Health Organisation (WHO Science Council, 2022). In that sense, the GLAD Project is a good start to increase representation of LatAm population in control databases that will help filter common variants in this population, but rare for the rest of the world. Also, similar regional networks and shared resources will need to be built for LatAm people with both common and rare diseases, following the example of to the Human Heredity and Health in Africa (H3Africa) consortium (Mulder et al., 2018). 

Apart from the need of growing genomic capabilities, the use of tailored model organisms is also necessary to help discovery and understanding of novel genetic diseases in LatAm. Even with the implementation of cutting-edge genomic resources, many patients with infrequent conditions (or novel syndromes) undergo frustrating journeys to obtain an accurate diagnosis. For low- and middle-incomes countries in LatAm, this milestone is far to be tackled. Studying human genomic variants in model organisms can bring substantial value to human disease research, especially for novel or infrequent conditions. Successful projects in high-incomes countries such as the UDN and the MOSC (Splinter et al., 2018), the Centers for Mendelian Genomics (CMG) (Baxter et al., 2022) and the Canadian Rare Diseases Models and Mechanisms Network (RDMM) (Boycott et al., 2020) provide remarkable paths for LatAm human geneticists, clinicians, and model organism researchers to collaborate seeking to determine pathogenicity of disease-linked variants in both novel and known genes, gain pathophysiological understanding of disorders, and even discover treatments (Strynatka et al., 2018). Lessons learned from these projects should encourage LatAm countries to work together to establish tailored frameworks for facilitating the diagnosis of local, and potentially new, syndromic disorders.

Apart from tackling aforementioned challenges, collaboration amongst clinicians and scientists should not be limited to LatAm countries with the required infrastructure to perform the whole process of NGDA, but it needs to be expanded to more underserved countries within the region. Furthermore, views and perspectives from people with rare diseases and their families as well as from indigenous populations must be included in this collaboration in order to make long-lasting networks. When these two last considerations are taken into account, then LatAm will lead the path to novel GDA discoveries in the world.
